# Frequency of Mutacin Gene Types in Streptococcus mutans Isolated From Oral Potentially Malignant Disorder (OPMD) Patients

**DOI:** 10.7759/cureus.66335

**Published:** 2024-08-06

**Authors:** Srudhika Sakthivel, A. S. Smiline Girija, Vijayashree J Priyadharsini, Kannika K Parameshwari

**Affiliations:** 1 Department of Microbiology, Saveetha Dental College and Hospitals, Saveetha Institute of Medical and Technical Sciences (SIMATS) Deemed University, Chennai, IND

**Keywords:** oral health, mutacin, health, caries, opmd, streptococcus mutans

## Abstract

Objectives

Mutacins are potent virulent factors attributing to the virulence in *Streptococcus mutans* leading to oro-dental diseases, and oral potentially malignant disorders (OPMDs) are considered a premalignant condition of the oro-mucosal layers in the oral cavity. The purpose of this study was to phenotypically characterize *S. mutans* from the clinical samples of patients with OPMD and to assess the frequency of mutacin genes in comparison with healthy individuals.

Methods

Saliva samples (n=60) were collected from three different groups and the samples were incubated at 37°C for 48 hours in Mutans-Sanguis agar. After incubation, the isolates were identified phenotypically for *S. mutans* and the frequency of mutacin genes and its types were assessed by polymerase chain reaction (PCR).

Results

*S. mutans* was found to be more prevalent in the OPMD cases (45%) followed by healthy individuals with caries (15%). Mutacin genes were expressed in all the groups except Group 3 (healthy individuals) without caries. Mutacin I was expressed the highest in Group 1 and Group 2 with 88% and 62.5, respectively, and mutacin III was expressed the least in all groups with 0% expression.

Conclusion

The findings of the study show the presence of mutacin gene types in the clinical strains of *S. mutans* in association with OPMD and caries. Further experimental evidence may be required to assess the frequency and to design a novel drug targeting the same.

## Introduction

Oral cancer is one of the most common malignant conditions worldwide and is considered the sixth and 12th most common cancer in men and women, respectively. Oral cancer usually occurs due to the progression or proliferation of oral potentially malignant disorders (OPMDs) or lesions [1]. An OPMD is defined as an abnormal epithelial lesion or a disorder in the mucosal layers of the oral cavity leading to malignant transformation. They are lesions that precede the onset of oral invasive cancers [2]. An OPMD is usually diagnosed as leukoplakia, which is a white lesion; in rare cases, it can be diagnosed as erythroplakia, which appears as a red lesion [3]. Other forms of early OPMD lesions include lichen planus and oral submucous fibrosis. OPMDs have an accelerated or increased risk of progression to cancer and the risk depends on a variety of patient-related or lesion-related factors. Some of these factors include site and type of lesion, gender, and habits such as alcoholism and chronic smoking [4]. The evolution of changes in the oral cavity in an OPMD patient includes changes in the color and thickness of the oral mucosa [5]. Understanding the etiology, clinical features, and appropriate management of OPMDs is crucial in ensuring timely intervention and reducing the risk of malignant transformation.

More than 700 species of bacteria or phylotypes have been discovered in the oral cavity and most of them are considered functional biomes [6]. These bacterial species are usually present in the oral cavity sites like the dorsum of the tongue, tonsils, vestibules, lateral sides of the tongue, hard and soft palate, buccal epithelium, and most importantly, supragingival and subgingival plaque accumulation on the tooth surface [7]. The most common species belong to *Veillonella, Granulicatella, Gemella*, and *Streptococcus* genera [8]. *Streptococcus mutans* possess an additive effect on the development and progression of oral cancer by increasing the aggressiveness of the infection, the mesenchymal transition of the epithelium, and by increasing the production and release of Interleukin (IL)-6 [9].

Some of the virulence factors of *S. mutans *involved in the pathogenesis are adhesion and cohesion during dental biofilm formation, acidogenicity, acid tolerance, and genes like *gbpA *and mutacins. Mutacin genes play an important role in the transmission and colonization of the bacteria and thus are considered the major virulence factor in *S. mutans *leading to human dental caries [10]. There are many types of mutacins, the major types being mutacin I, mutacin II, mutacin III, and mutacin IV. These mutacins play a major biological role in the colonization of *S. mutans *strains, mainly type I [11].

Literature reviews have documented *S. mutans* and their association with dental caries and other diseases, but very limited literature exists on the role of mutacin genes of *S. mutans* in OPMD patients. This study is thus aimed to determine and assess the frequency of mutacin gene types in *S. mutans* from OPMD and caries-affected cases.

## Materials and methods

This study was conducted at the Department of Microbiology, Saveetha Dental College and Hospitals, Chennai, Tamil Nadu, India. The study was approved by the Institutional Human Ethical Committee - Saveetha Dental College and Hospitals (approval number: SRB/SDC/UG-2141/23/MICRO/125, IHEC/SDC/UG-2141/23/MICRO/275).

Sampling data

Saliva samples (n=60) were collected from 20 patients at the Department of Oral Medicine and Radiology, Saveetha Dental College and Hospitals, and divided into three different groups: Group 1 (OPMD- conditions of submucous fibrosis, leukoplakia, and any other abnormal lesions), Group 2 (Healthy patients with caries), and Group 3 (Healthy individuals without caries). The samples were streaked on the sterile Mutans-Sanguis agar and the plates were incubated at 37°C for 48 hours. The colonies were observed by Gram staining and by negative catalase test.

Isolation and identification of *S. mutans*

The saliva samples from the study population were collected in sterile containers and were immediately transferred to the microbiology laboratory. The samples were streaked onto the sterile Mutans-Sanguis agar which contains specific ingredients for the growth of *S. mutans* and the plates were incubated at 37°C for 48 hours. After incubation, the colonies were observed by Gram staining for gram-positive cocci in short chains. Phenotypic characterization was done by using a negative catalase test, negative optochin sensitivity, and negative bile esculin test. 

Molecular detection of mutacin types

Genomic DNA was extracted from the fresh broth suspension of the isolates using the manufacturer’s instructions (QIAGEN N.V., Hilden, Germany) and polymerase chain reaction (PCR) was performed using the specific primers (Table 1).

**Table 1:** Primers used in polymerase chain reaction analysis of mutacin I, II, III and IV genes in Streptococcus mutans

Mutacin type	Primers	Annealing temperature	Amplicon size
Mutacin I and III	5’-AGTTTCAATAGTTACTGTTGC-3’ 5’-GCCAAACGGAGTTGATCTCGT-3’	55°C	444 bp
Mutacin II	5’-AACGCAGTAGTTTCTTTGAA-3’ 5’-TTCCGGTAAGTACATAGTGC-3’	55°C	450 bp
Mutacin IV	5’-ATGGGATATTTAAAGGGAAA-3’ 5’-TCAGAGCAGCTACAAAAACT-3’	55°C	720 bp

A 15 μl of the amplification reaction mixture was prepared using double-distilled water (5.6 μl) and 2× Master Mix (7.8 μl) (Takara Bio Inc., Kusatsu, Shiga, Japan). In a thermocycler (Mastercycler; Eppendorf, Hamburg, Germany), 36 cycles of amplification were performed using the specific primers for mutacin types. Confirmation of the PCR amplicons was done using a 100-bp DNA ladder and visualization was done in agarose gel electrophoresis (1%).

## Results

Isolation and identification of S. mutans

**Figure 1:**
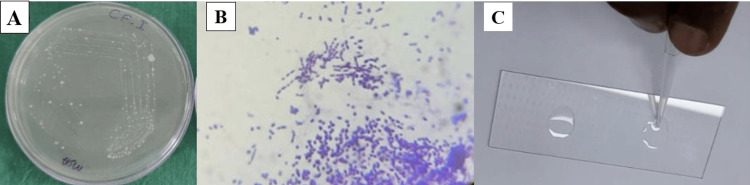
Characterization of Streptococcus mutans from the OPMD patients. (A) Colony morphology of S. mutans in Mutans-Sanguis agar plate; (B) Gram staining showing gram-positive cocci in short chains; (C) Negative catalase test. OPMD: oral potentially malignant disorder

S. mutans formed a characteristic frosted glass appearance on the Mutans-Sanguis agar with adhesive colonies with typical gram-positive cocci with a negative catalase test (Figure 1).

It was highly prevalent in the OPMD cases (45%; n=9) followed by healthy patients with caries (40%; n=8), and least in the healthy individual without caries (15%; n=3) (Table 2).

**Table 2:** Frequency of Streptococcus mutans and mutacin gene types among the study groups

Groups	Streptococcus mutans	Mutacin I	Mutacin II	Mutacin III	Mutacin IV
Group 1 (OPMD patients) (n=20)	9 (45%)	8 (88%)	2 (22%)	-	4 (44%)
Group 2 (Healthy Controls With Caries) (n=20)	8 (40%)	5 (62.5%)	-	-	1 (12.5%)
Group 3 (Healthy Controls Without Caries) (n=20)	3 (15%)	-	-	-	-

Molecular detection of mutacin gene types

**Figure 2:**
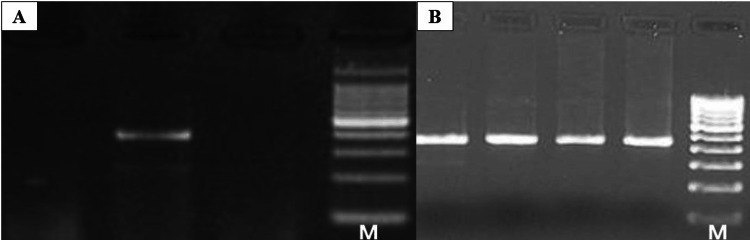
Electrophoretogram of (A) mutacin I/III genes (amplicon size 444 bp) and (B) mutacin II gene (amplicon size 450 bp) with standard DNA marker ladder; Lane M = 100 bp DNA marker

Among the four mutacin gene types screened in the OPMD cases (Group 1), mutacin type I was detected in higher frequency (88%; n=8), followed by type II (22%; n=2) and type IV (44%; n=4). In healthy patients with caries (Group 2), mutacin type I was most prevalent (62.5%) followed by type IV in a single isolate. In the healthy individuals without caries (Group 3), no mutacin types were observed from the three isolates. In all the three groups under study, mutacin type III was not present (Figure 2).

## Discussion

OPMD is considered a disorder of the oral epithelial layer with an increased risk for malignant transformation. The presence of epithelial dysplasia and its degree are the most useful indicators of malignant transformation risk [12]. The prevalence of OPMD differs based on the geographical habitat and other host factors [13]. Many risk factors such as the consumption of tobacco and areca nut and the presence of diabetes have a strong association and effect on OPMD malignant transformation [14]. Potential microbial pathogens were also involved in the progression and further complications in OPMD and caries cases. In this context, S. mutans possess abilities to produce acids and to adhere to the tooth surface which leads to their cariogenicity [15]. The present study is thus designed to screen for the prevalence of the S. mutans isolates in correlation with OPMD and diseased conditions. 

In the present investigation, S. mutans was more prevalent in OPMD cases (45%) followed by healthy individuals with caries (40%), and much less frequent in cases without caries (15%). This study correlates with an earlier study where S. mutans was less frequent in patients without caries substantiating the role of S. mutans with caries and in diseased conditions [16]. From this, we can infer that there exists a correlation between OPMD and S. mutans, due to its ability to adhere to OPMD lesions and to cause the spread of the infection by rapid colonization [17,18].

Mutacin gene is a bacteriocin produced by S. mutans attributing to the cariogenicity nature of the bacteria. They facilitate the colonization of the bacteria and its establishment in dental biofilm [19]. There is no literature stating the association between mutacin genes and OPMD, which is the focus of this study. In recent years, many studies have been documented on the virulence of S. mutans. A study done on the isolation of mutacin genes from S. mutans strains from caries-free and caries-active individuals concluded that mutacins play a vital role in colonization by S. mutans strains, mainly in the niche of high-complexity microbial communities [11]. The PCR analysis in the same study showed 31.8% of mutacin IV genes in caries-free individuals and did not detect mutacin I/III and mutacin II in both groups, while in the present study, mutacin IV was not detected in caries-free individuals and we were able to isolate mutacin I/IV in almost all the groups. S. mutans are found in high numbers of OPMD cases and individuals with dental caries, so ideally, we were able to isolate the mutacin genes in OPMD patient strains and in strains of healthy individuals with caries. The expression of mutacins genes was relatively lesser in healthy individuals without caries. Since dental caries and OPMD are rapidly increasing dental conditions, continuous and periodical research must be done to isolate and study multiple virulent factors including genes associated with them.

There is little to no data available for comparison, which poses a limitation to the findings. Statistical analysis was not possible due to the small sample size, notwithstanding the possibility of finding distortion. Predicting the outcomes on a bigger scale may also be hampered by the small sample size. This study evaluated the frequency of mutacin genes in S. mutans present in OPMDs solely, without considering the association with other types of virulence factors, despite being the most prominent bacterium. Also, periodical monitoring of virulence genes will aid in the control of S. mutans in patients with OPMD. S. mutans being prevalent in all dental operatories [20,21], it is also the need of the hour to survey such strains in all dental healthcare setups. Putative vaccine candidate predictions for priority pathogens have sparked interest in recent years and are achievable by computational platforms [22,23]. Mutacins may be thus considered as a novel target in designing a caries vaccine.

## Conclusions

*S. mutans* and mutacins were highly associated with OPMD and cases with caries, substantiating their potential role in the complication of the disease. This is the first-of-its-kind study to evaluate the types of mutacins in S. mutans isolated from OPMD and caries cases. Thus, dental research may be further initiated targeting mutacin-based genetic determinants in order to combat the complications caused by S. mutans in association with OPMD and caries. Also, mutacins and their types may be considered vital targets, both for rapid diagnosis and also toward a theranostic approach.

## References

[1] Mortazavi H, Baharvand M, Mehdipour M (2014). Oral potentially malignant disorders: an overview of more than 20 entities. J Dent Res Dent Clin Dent Prospects.

[2] Warnakulasuriya S (2018). Clinical features and presentation of oral potentially malignant disorders. Oral Surg Oral Med Oral Pathol Oral Radiol.

[3] Wetzel SL, Wollenberg J (2020). Oral potentially malignant disorders. Dent Clin North Am.

[4] Speight PM, Khurram SA, Kujan O (2018). Oral potentially malignant disorders: risk of progression to malignancy. Oral Surg Oral Med Oral Pathol Oral Radiol.

[5] Warnakulasuriya S (2020). Oral potentially malignant disorders: a comprehensive review on clinical aspects and management. Oral Oncol.

[6] Girija AS, Ganesh PS (2022). Functional biomes beyond the bacteriome in the oral ecosystem. Jpn Dent Sci Rev.

[7] Rajasekar A, Varghese SS (2022). Microbiological profile in periodontitis and peri-implantitis: a systematic review. J Long Term Eff Med Implants.

[8] Aas JA, Paster BJ, Stokes LN, Olsen I, Dewhirst FE (2005). Defining the normal bacterial flora of the oral cavity. J Clin Microbiol.

[9] Tsai MS, Chen YY, Chen WC, Chen MF (2022). Streptococcus mutans promotes tumor progression in oral squamous cell carcinoma. J Cancer.

[10] Kamiya RU, Taiete T, Gonçalves RB (2011). Mutacins of Streptococcus mutans. Braz J Microbiol.

[11] Kamiya RU, Napimoga MH, Höfling JF, Gonçalves RB (2005). Frequency of four different mutacin genes in Streptococcus mutans genotypes isolated from caries-free and caries-active individuals. J Med Microbiol.

[12] Farah CS, Woo SB, Zain RB, Sklavounou A, McCullough MJ, Lingen M (2014). Oral cancer and oral potentially malignant disorders. Int J Dent.

[13] Mello FW, Miguel AF, Dutra KL, Porporatti AL, Warnakulasuriya S, Guerra EN, Rivero ER (2018). Prevalence of oral potentially malignant disorders: a systematic review and meta-analysis. J Oral Pathol Med.

[14] Singh AK, Chauhan R, Anand K, Singh M, Das SR, Sinha AK (2021). Prevalence and risk factors for oral potentially malignant disorders in Indian population. J Pharm Bioallied Sci.

[15] Hamada S, Koga T, Ooshima T (1984). Virulence factors of Streptococcus mutans and dental caries prevention. J Dent Res.

[16] Hillman JD (2002). Genetically modified Streptococcus mutans for the prevention of dental caries. Antonie Van Leeuwenhoek.

[17] Beachey EH (1981). Bacterial adherence: adhesin-receptor interactions mediating the attachment of bacteria to mucosal surface. J Infect Dis.

[18] Hall-Stoodley L, Costerton JW, Stoodley P (2004). Bacterial biofilms: from the natural environment to infectious diseases. Nat Rev Microbiol.

[19] Merritt J, Qi F (2012). The mutacins of Streptococcus mutans: regulation and ecology. Mol Oral Microbiol.

[20] Malini M, Thomas TK, Bhargava D, Girija S (2012). Microbiology of the white coat in a dental operatory. Indian J Dent Res.

[21] Smiline GA, Pandi SK, Hariprasad P, Raguraman R (2012). A preliminary study on the screening of emerging drug resistance among the caries pathogens isolated from carious dentine. Indian J Dent Res.

[22] Smiline Girija AS (2020). Delineating the immuno-dominant antigenic vaccine peptides against gacS-sensor kinase in Acinetobacter baumannii: an in silico investigational approach. Front Microbiol.

[23] Sankar S (2022). In silico design of a multi-epitope chimera from Aedes aegypti salivary proteins OBP 22 and OBP 10: a promising candidate vaccine. J Vector Borne Dis.

